# PREDICTORS OF HOSPICE AND END-OF-LIFE CARE: GEOGRAPHIC LOCATION, PATIENT PORTALS, NURSING HOME CARE, AND HOME HEALTH

**DOI:** 10.1093/geroni/igad104.0243

**Published:** 2023-12-21

**Authors:** Jennifer Portz, Karla Washington

## Abstract

Hospice is considered an indicator of high-quality care at end-of-life (EOL) often allowing patients to receive care aligned with their values, in their homes, surrounded by support and loved-ones. However, there is a need to address access barriers to hospice and enroll patients earlier in their disease trajectory. Understanding predictors of hospice use will help identify strategies for addressing hospice inequities, promoting longer hospice stays, and improving overall EOL outcomes. Focusing on research methodologies with large cohorts and national samples, this symposium examines the associations of health care services and inequalities on key EOL outcomes and hospice use. First, Autumn Decker will describe profiles of hospice use and EOL experience by geographic location and race from the National Health and Aging Trends Study. Next, Dr. Jennifer Portz will summarize the association between types of patient portal use and EOL outcomes including hospitalization, advance directives, and hospice use. Dr. Ellis Dillon will follow with a description of hospice use and short hospice length of enrollment for Conneticut’s Medicaid decedents, comparing decedents with nursing home stays to those without. Lastly, Dr. Olga Jarrin will present findings from her study of home health care use during the last 3 years of life as a predictor of death with hospice in a Medicare cohort. Our discussant, Dr. Karla Washington, will lead us in a conversation outlining the role of health care services, such as home health, nursing home care, and patient portals, for promoting access to hospice earlier and for all patients.

